# Supplementary material for: Association between oral microbiome and seven types of cancers in East Asian population: a two-sample Mendelian randomization analysis
Source: Front Mol Biosci. 2023 Nov 21;10:1327893. doi: 10.3389/fmolb.2023.1327893 (PMC10702768; doi:10.3389/fmolb.2023.1327893)
Supplement: Supplementary file 1 [file DataSheet1.ZIP › Supplementary Materials/MR plots for tongue/tongue═╝/Prostate cancer/pheno.1905_to_prostate cancer_leave_one_out.pdf]

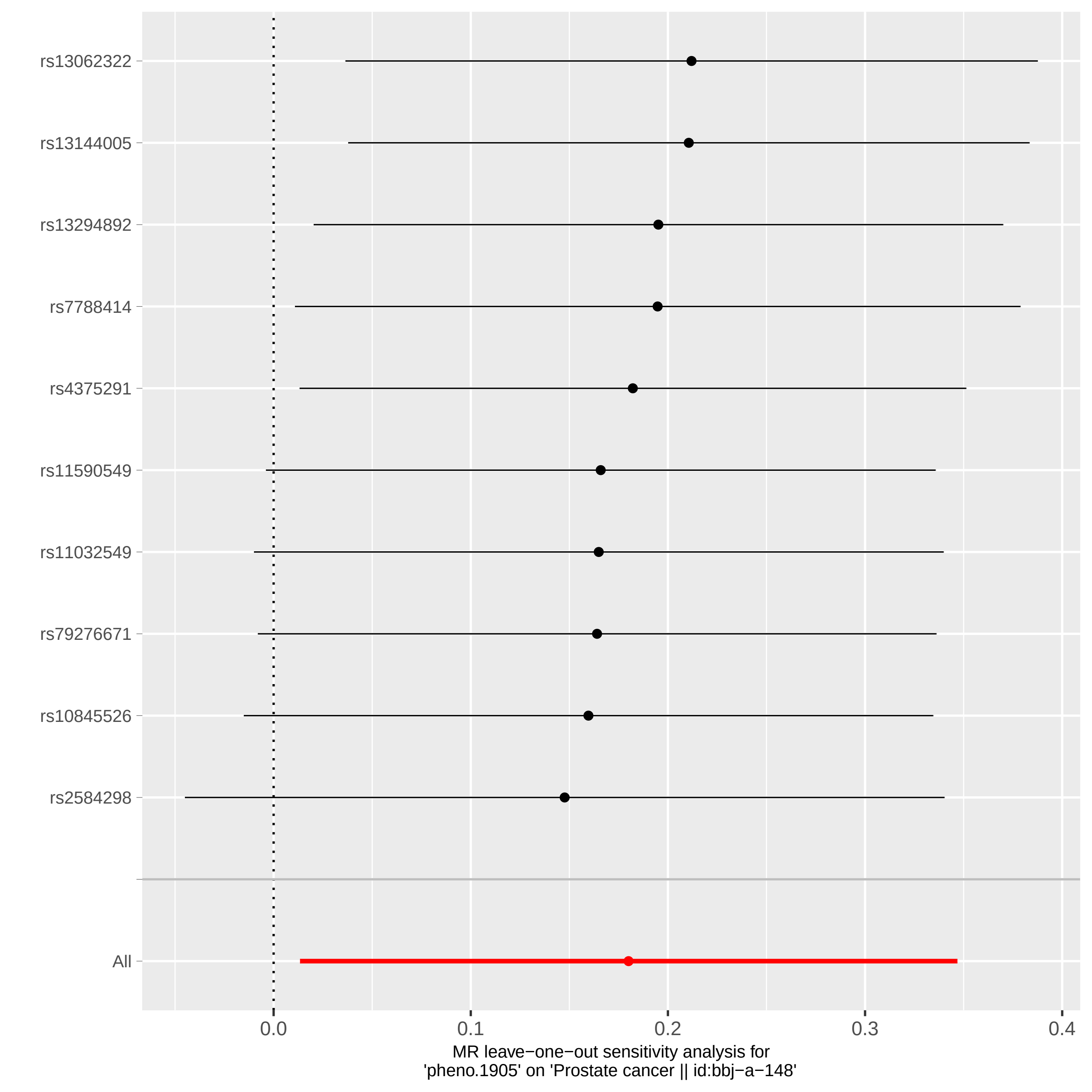
rs13062322
rs13144005
rs13294892
rs7788414
rs4375291
rs11590549
rs11032549
rs79276671
rs10845526
rs2584298
All
0.0
0.1
0.2
0.3
0.4
MR leave−one−out sensitivity analysis for
'pheno.1905' on 'Prostate cancer || id:bbj−a−148'
